# Iron deficiency and fatigue in inflammatory bowel disease: A systematic review

**DOI:** 10.1371/journal.pone.0304293

**Published:** 2025-01-13

**Authors:** Stephanie Sartain, Maryam Al-Ezairej, Martin McDonnell, Catherine Westoby, Vasiliki Katarachia, Stephen A. Wootton, J. R. Fraser Cummings

**Affiliations:** 1 Department of Gastroenterology, University Hospital Southampton NHS Foundation Trust, Southampton, United Kingdom; 2 University Hospitals of Leicester NHS Trust, Leicester, United Kingdom; 3 School of Human Development and Health, Faculty of Medicine, University of Southampton, Southampton, United Kingdom; Karolinska Institutet Institutionen for medicinsk epidemiologi och biostatistik, SWEDEN

## Abstract

**Background:**

It is unclear what impact iron deficiency has on fatigue in people with inflammatory bowel disease (IBD). This systematic review examined the evidence of whether iron deficiency, with or without anaemia, was associated with fatigue in IBD. Fatigue is a common symptom in patients with IBD that can be difficult to manage and treat. A greater understanding of the role and contribution of iron deficiency to fatigue may help improve the management of this condition.

**Methods:**

The databases searched were MEDLINE, OVID, CINAHL and Web of Science. Inclusion criteria were studies measuring iron status for iron deficiency (ID) and patient-reported outcome measures (PROMs) for fatigue in patients with IBD of any level of disease activity. Assessment of bias was conducted using the Newcastle Ottawa Scale. Studies were grouped for syntheses according to whether exposure was iron deficiency without anaemia (IDWA) or ID regardless of haemoglobin level.

**Results:**

Two hundred and eighty-five individual database results were identified and screened; 32 complete records were reviewed, from which seven studies with 1425 individuals were deemed eligible for inclusion in the results synthesis. Considerable variation in the methods and statistical analysis used to investigate the relationship between ID and fatigue prevented any quantitative synthesis. Studies varied by population disease activity levels, approaches used to define ID and PROMs used to measure fatigue. Three studies directly compared fatigue scores in IDWA to those not iron deficient, two of which showed patients with IDWA had significantly lower fatigue scores. Four studies used ID irrespective of anaemia as the exposure and reported mixed results on fatigue, with only one study reporting a higher prevalence of fatigue in the ID group.

**Conclusions:**

There was marked heterogeneity between studies in this review. Two studies found evidence of a slight increase in fatigue levels in patients with IDWA. Though this does not explain all fatigue in patients with IBD, iron replacement should be considered to improve fatigue in iron-deficient patients.

## Introduction

Fatigue is a frequent manifestation in patients with inflammatory bowel disease (IBD), negatively impacting health-related quality of life, social functioning and employment [1, 2]. It is highly prevalent in active disease, with 72% of patients affected, but it also affects 47% of those in remission [3]. The cause of fatigue in inflammatory bowel disease is currently not well understood. It is associated with active disease, poor sleep, anaemia, increased anxiety and depression [3, 4], indicating multifactorial causality. At this time, there are few evidence-based treatment options or interventions available to improve fatigue in IBD. Iron deficiency is associated with fatigue in other populations and may represent a treatable cause of fatigue in patients with IBD.

Iron is essential for oxygen transport by haemoglobin and generating energy in the mitochondria; thus, reduced availability leads to fatigue. Deficiency will lead to decreased haemoglobin levels and the development of anaemia. Iron deficiency can occur without anaemia, known as iron deficiency without anaemia (IDWA). Significant improvements in treatments for iron deficiency have been made, including better-tolerated oral preparations [5] and safer parenteral iron preparations [6]. Treating iron deficiency is associated with improvements in fatigue in healthy populations, including those who are not anaemic [7–9].

Active inflammation in IBD predisposes to iron deficiency due to iron losses from bleeding in the gut and hepcidin-mediated inhibition of iron absorption and sequestration of body iron stores. In recent studies, the prevalence of iron deficiency in IBD varies between 23.7–43.4% [10, 11]. There has been a greater focus in IBD on the relationship between iron deficiency or anaemia and quality of life rather than fatigue. Replacing iron in patients with IDA improves QOL [12–15], as does iron replacement in IDWA [16, 17]. Improvements in fatigue levels may explain increases in quality of life after iron replacement though studies examining the relationship between fatigue and iron deficiency in IBD show mixed results [18–24]. The presence of anaemia may complicate interpreting the effect of iron deficiency on fatigue, as anaemia is associated with fatigue in many populations, though the evidence in IBD is mixed [18, 25–27]. There is a lack of well-designed studies investigating the effects of iron replacement on changes in fatigue in patients with IBD. A subgroup analysis of patients with IBD included in a cohort of gastroenterology patients receiving ferumoxytol versus placebo for IDA showed significant improvements in fatigue PROM scores in the treatment group [28]. A small study showed improvements in fatigue, haemoglobin and transferrin saturations in patients with IBD and mild anaemia receiving liposomal iron [29].

The definition of fatigue and how it is measured contribute to the challenges in understanding this condition. Fatigue can be differentiated into physical and mental components. Most studies in the current literature use patient-reported outcome measures (PROMs) to investigate overall fatigue in IBD. Several biochemical parameters, such as plasma ferritin and transferrin saturation, can define iron deficiency. These have limitations in inflammation, and the cut-off values applied to indicate iron deficiency vary. All these factors can affect the interpretation of the existing evidence.

Understanding the role and contribution of iron deficiency, with and without anaemia, as a determinant of fatigue in IBD will improve the management of this condition. This review aimed to examine the evidence from the published literature on whether iron deficiency in people with IBD is associated with fatigue. The results will improve fatigue management in patients with IBD by understanding the need to detect and treat iron deficiency.

## Methods

### Registration

This review was registered with PROSPERO on 25/11/2022, ID CRD42022368406. Due to differences in experimental design, study population, outcome measures, and statistical analysis methods, a meta-analysis of the included studies was impossible. Therefore, the Synthesis Without Meta-analysis (SWIM) guidelines [30] were followed for data synthesis.

PICO and eligibility criteria (Table 1)

**Table 1:** PICO and eligibility criteria.

PICO	Inclusion criteria	Exclusion criteria
Population	Adult patients with inflammatory bowel disease	Non-IBD populations, patients under 18 years
Exposure	Iron deficiency measured in keeping with guidelines	Iron deficiency not measured in keeping with guidelines
Comparison	Normal iron status	No comparison of iron status
Outcome	Self-reported measures of fatigue	Other outcomes such as quality of life

This review sought to determine whether patients with IBD and iron deficiency experience worse self-reported fatigue than non-iron deficient IBD patients. Studies considered eligible included adult subjects with IBD, where blood tests were taken to measure iron status and fatigue, measured by patient-reported outcome measures. Studies with any level of IBD activity were included. The definition of iron deficiency used and the parameters measured must be in keeping with recognised guidance, such as ECCO guidelines from 2015 on anaemia [31]. There was no restriction on the type of study included.

### Information sources and search strategies

The databases searched were Medline, Embase, CINAHL and Web of Science Core Collection between 22/11/2022 and 22/05/23. These were limited to English-language studies and for adult participants aged over 18 years. The search terms “inflammatory bowel disease”, “iron deficiency”, and “fatigue” were used with the Boolean operator “AND” to combine them. Words related to these were also searched using the Boolean operator “OR”, for example, the different types of IBD, Crohn’s disease and ulcerative colitis (UC). See S1 File for each database search strategy. Grey literature was also searched using Proquest. The references in the papers reviewed were checked using the “snowballing” approach.

### Study selection process

The database search results were uploaded to Rayyan [32], and duplicates were removed. Two reviewers (SS and MA-E) independently screened the abstracts. Full papers were retrieved for abstracts that appeared to meet eligibility criteria. Both reviewers reviewed these independently to decide if they met the inclusion criteria. In the case of disagreement, the opinion of a third reviewer (SAW) was sought.

### Data extraction

Data was extracted from each study by one reviewer (SS) and confirmed by a second reviewer (MA-E). The variables included were number of participants, diagnosis of participants, age included, measures of disease activity used, inclusion criteria for disease activity, parameters used to define iron deficiency, PROMs used to measure fatigue and results data. Outcome data collected depended on the study design and how results data was presented, such as mean fatigue scores or proportion of fatigued patients that were iron deficient. Missing or unclear data was recorded as such.

### Risk of bias

Two reviewers independently assessed the risk of assessment bias using the Newcastle-Ottawa score adapted for cross-sectional studies (see S2 File). A third reviewer was sought to determine any differences in opinion.

### Synthesis methods

Studies were prioritised to synthesise the main results according to the study design. The inclusion criteria were studies that identified iron-deficient patients with IBD and described their self-reported measures of fatigue or the proportion of fatigued patients compared to patients who were not iron-deficient. As a result, studies in the main results synthesis contained a group with definite iron deficiency exposure. The main results of the synthesis excluded studies that presented the iron status of fatigued patients compared to non-fatigued patients or other methods. This was due to limitations in determining iron status and the lack of a group exposed to iron deficiency. Studies were sub-grouped into two groups to synthesise the main results, according to whether the exposure was IDWA or iron deficiency with any haemoglobin level. As anaemia is associated with fatigue, it may be a potential confounder. This was a deviation from the planned protocol, where studies were due to be grouped by study design and outcome measures, such as analysing the average fatigue score for iron deficiency vs. non-iron deficiency. A wide variety of methods and outcome measures were used in the studies, and grouping was restricted in this manner.

The main results synthesis for each of the two subgroups were presented in tabular format containing the data from the studies included. The findings of each study were displayed as results data in the form that was extracted from the papers included. Due to the variation in methods and outcomes reported, it was not feasible to convert the results to a standardised metric of effect measures or combine them in summary statistics. Instead, the direction of effect from each study was displayed in the synthesis tables so a vote-counting approach could be undertaken. Studies which reported the results of their analysis without providing the results data were included in this approach. Key study characteristics that may affect the interpretation of the data were also included in the results tables, such as the disease activity levels of patients included in each study. This will demonstrate the subgroups’ heterogeneity in reported effects when synthesising the main results. Tables were structured according to the risk of bias score in each subset. The certainty of evidence was assessed using a GRADE approach for systematic reviews without a single estimate of effect (Murad 2017).

## Results

**Fig 1:**
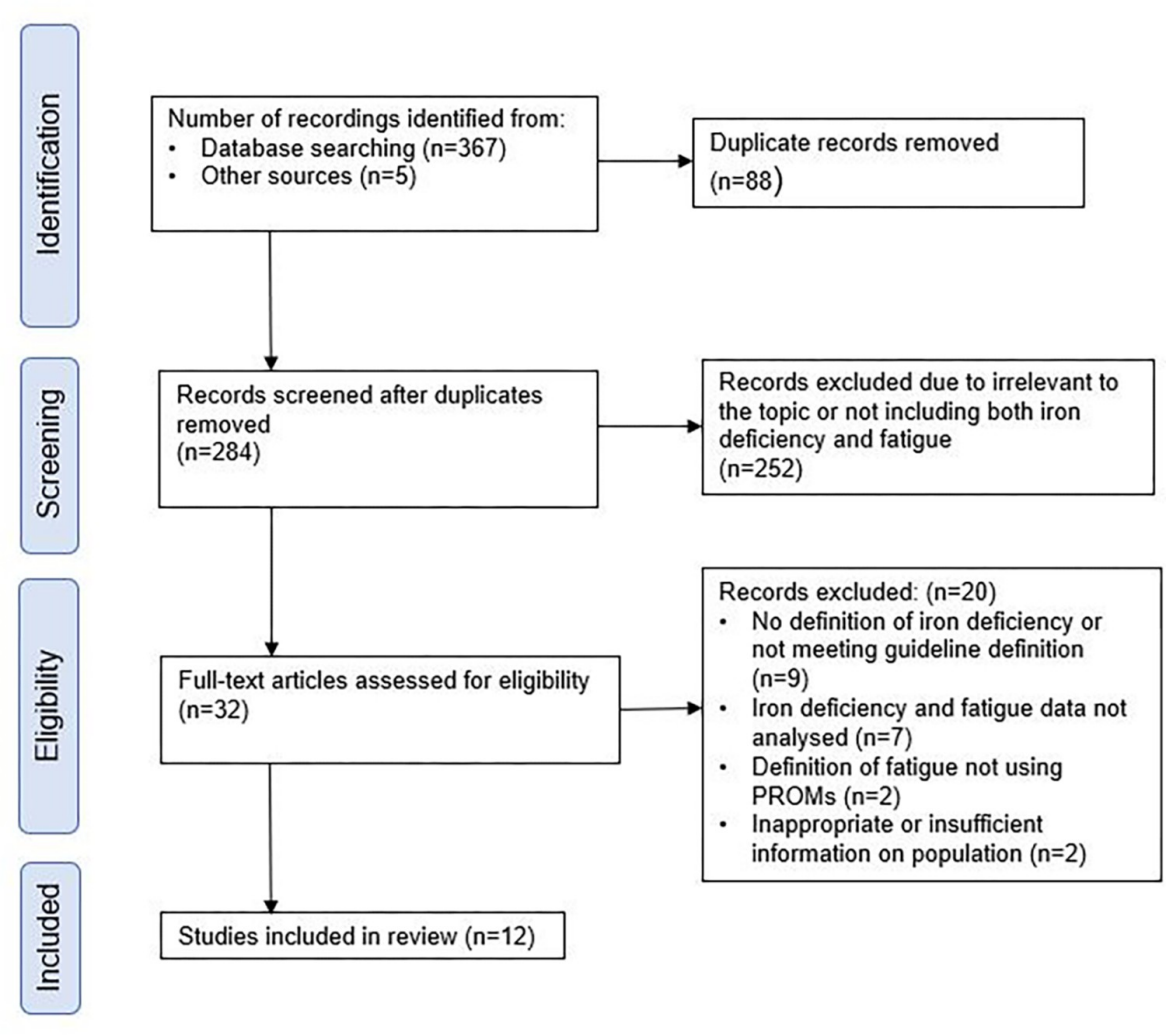
Prisma flow diagram.

Three hundred and seventy-two results were identified from database searches or other sources. After removing 87 duplicates, the remaining 284 abstracts were screened, and 32 complete records that appeared to meet the eligibility criteria were reviewed. S3 File lists the 284 records that were identified and reasons for exclusion for those not included in the review. Eighteen studies measured both iron status and fatigue PROMs in patients with IBD. However, the methods of defining iron deficiency and the PROMs used varied considerably between studies. Twelve studies measured iron status in keeping with guidelines, so they were deemed eligible. Studies that gave iron replacement were not included as there was no analysis of fatigue levels by iron status in individuals in these studies [5, 29]. Fig 1 shows the Prisma flow diagram. The remaining 12 studies that were included are outlined in the study characteristics Table 2. These contained 2465 patients.

**Table 2:** Characteristics of included studies.

Study	Population	Number	Type of study	Primary aim of study	Disease activity and inclusion	Methods	Exposure: ID or IDWA	Definition of ID	Fatigue PROMs	ID associated with fatigue
Aluzaite et al, 2019 [23]	Adult IBD > 18 years	113 UC: 43, CD: 79 47.8% (n = 54) had ID	Prospective, observational, cross-sectional, single centre, outpatients	Determine prevalence of fatigue in IBD patients and investigate associated variables.	Active (n = 70) Remission(n = 43) Active = ≥1 of: CDAI>150, SCCAI>5, FCP>150μg/g, CRP >5mg/L. 70% of CD and 49% of UC had active disease	Comparing mean fatigue scores in iron deficient v non-iron deficient patients.	ID	ferritin < 30μg/L or <100μg/L in inflammation (as defined by ECCO 2015)	1.MFI: each dimension scored 4–20. >10 = moderate fatigue and > 14 = severe fatigue. Overall >50: mod to severe. >70: severe fatigue 2.BFI >4 = moderate fatigue, >6 = severe fatigue	No
Bager et al, 2012 [18]	Adult IBD	425 UC: 174, CD: 251	Prospective, observational, cross-sectional, multicentre, outpatients	Prevalence of fatigue and the determinants of fatigue in IBD outpatients	Any disease activity. Active disease defined by HBI >4, SCCAI > 4.	Comparing mean fatigue scores in iron deficient v non-iron deficient patients.	ID	ferritin < 30μg/L or <100μg/L in inflammation (CRP above upper ref range)	MFI-20–5 dimensions of fatigue Score of 4–20 in each dimension. Higher score is more fatigue	No
Banovic et al, 2011 [33]	Adult IBD	81 CD: 59 UC: 22	Prospective, observational, single centre, outpatients	Determine the relationship between personality and IBD related fatigue in remission.	Remission only. CD HBI <5, UC Lichtiger Index < 7. Patients selected for reporting fatigue	Comparing mean ferritin levels in patients with high v low fatigue scores	ID	Ferritin and CRP measured—no further info given about adjusting ferritin levels in inflammation.	MFI global score: high fatigue MFI > 43, low fatigue MFI ≤43	No
Chavarria et al, 2019 [34]	Adult IBD > 18 years	544 CD: 332	Multicentre, observational, prospective study	Determine the prevalence of fatigue, factors associated with fatigue severity, the impact of fatigue on QOL, the relationship between fatigue and sleep disorders.	Any disease activity. Active disease in CD: HBI > 4, in UC partial Mayo ≥ 2.	Comparing mean ferritin levels in patients with high v low fatigue scores	ID	Anaemia: Hb <13g/dL in males and <12g/dL in females. ID = ferritin < 30μg/L without inflammation and < 100μg/L with inflammation. Not clear if ferritin cut off used in analysis.	Fatigue Severity Scale: fatigue = score ≥5 used in analysis categories. Fatigue Impact Scale: no cut off given	No
Goldenberg et al, 2012 [22]	Adult IBD > 18 years	280 CD: 143 UC: 137 230 not anaemic	Prospective, observational, cross-sectional from Manitoba cohort	To investigate the relationship between iron deficiency and fatigue in IBD in the absence of anaemia.	active and remission. 128/280 (46%) have active disease, defined as HBI or Powell Tuck ≥ 5	Comparing mean fatigue scores in iron deficient v non-iron deficient patients, all without anaemia	IDWA	IDWA: Anaemia Hb < 140g/dL in males, < 120g/dL in females, ferritin < 20ug/L or STR > 28mg/L	MFI 5 domains. General Fatigue subscale ≥ 13 = significant fatigue	No.No significant difference in MFI domains. No significant difference in IDWA prevalence in fatigued patients.
Gonzalez Alayon et al, 2017 [21]	Adult IBD age 18–70 years	127 CD: 78 UC: 49 16 anaemic patients removed from analysis	Prospective, observational, cross-sectional, single centre	Determine the prevalence of IDWA in outpatient setting, the secondary outcome was impact of IDWA on HrQOL and fatigue.	Any disease activity. Inflammation present in 21.3%, defined as CRP > 5mg/L with symptoms of active disease or investigations showing inflammation.	Comparing fatigue scores in iron deficient v non-iron deficient patients. Prevalence of extreme fatigue FACIT F < 30.	IDWA	Anaemic Hb < 130g/dL in males, < 120g/dL in females. ID: Ferritin <30 ug/L, ferritin < 100 ug/L in inflammation (CRP <5mg/L and active disease) and TS <16%	FACIT-F	Yes
Grimstad et al, 2015 [35]	Adult IBD age > 16 years	81 CD: 21 UC: 60	Prospective, observational, single centre	Determine the prevalence of fatigue in a cohort of newly diagnosed IBD compared to matched healthy controls. Explore the associations of fatigue and depression and marker s of disease activity.	Newly diagnosed IBD. Inflammation = CRP ≥ 5mg/L and calprotectin ≥ 50μg/g	Associations with iron (ferritin) and fatigue score in linear regression.	ID	ID: ferritin < 30ug/L if CRP < 5mg/L and ferritin < 100 ug/L if CRP ≥5mg/L.	fVAS: ≥50mm on 100mm line indicates fatigue FSS: ≥4 indicates fatigue	Yes when adjusted for age and gender.
Herrera-deguise et al, 2016 [19]	Adult IBD	104 CD: 58 UC: 46	Prospective, observational, cross-sectional	Evaluate the influence of IDWA on HrQOL and fatigue in IBD in clinical remission.	Remission HBI or SCCAI <5, normal Hb and CRP < 5. Median HBI = 0, SCCAI = 0.5	Comparing fatigue scores in iron deficient v non-iron deficient patients.	IDWA	IDWA: ferritin <30 ug/L and TS < 16% and normal Hb <13g/dL in male and < 12g/dL in females	Fatigue Impact Scale: higher score indicates fatigue	Yes
Jonefjall et al, 2018 [20]	Adult UC	288 UC only	Prospective, observational, cross-sectional, outpatients, multicentre	Investigate prevalence of high fatigue and determine risk factors for fatigue in UC in remission and active disease	Active (n = 155) and remission (n = 133)	Comparing the prevalence of iron deficiency in high fatigue v no fatigue	ID	ID: Ferritin <30 ug/L with CRP < 5mg/L. In active disease: ferritin 30–100 ug/L with TS <16%/ raised sTFR or TS <20% and raised CRP.	MFI: General fatigue ≥13 = significant fatigue	Yes
Konig et al, 2020 [24]	Adult IBD age >18 years	88, CD: 70 UC: 28	Prospective, Observational, single centre, outpatients	To determine the influence of ID, anaemia and inflammation with regard to neurological symptoms such as QoS, fatigue, depression or anxiety.	Any disease activity	Comparing the prevalence of patients with fatigue in those who have ID v non-ID.	ID	Anaemia = Hb <12g/dL in females, <13g/dL in males, ID = ferritin <30ug/L or ferritin < 100 ug/L and TS < 20% if CRP >5mg/L.	Piper Fatigue Scale > 4 = fatigue	Tendency to fatigue
Truyens et al, 2021 [36]	Adult IBD	157 CD: 78.3% UC: 21.7%	Prospective, observational, single centre, tertiary	Determine the prevalence of fatigue in IBD in remission. Secondary objective was to report clinical and biochemical factors associated with fatigue.	Remission determined by HBI or clinical Mayo score and in biochemical remission (not defined). Low Hb excluded. see refs also	Multiple regression analysis of factors associated with fatigue using transferrin saturation for iron status.	ID	ID: ferritin < 30 ug/L or TS < 20%. Used TS for analysis	SFQ: fatigue ≥18 fVAS: fatigue ≥5	Yes
Villoria et al, 2017 [37]	Adult IBD > 18 years	177 CD 72% UC 28%	Prospective, observational, outpatients, single centre	Investigate biological factors associated with fatigue in IBD including micronutrients	Excluded if flare in last 3 months, HBI ≤ 5 in 90% and ≤ 7 in 98% CD, Mayo≤ 2 in 78%, ≤ 4 in 93% UC.	Comparing ferritin levels in 3 different levels of fatigue score	ID	No definition of iron deficiency given. Measured TS, ferritin, iron	FACIT-F	No

Abbreviations

BFI = Brief Fatigue Inventory, FACIT-F = Functional Assessment of Chronic Illness Therapy Fatigue, fVAS = Fatigue Visual Analogue Scale, HBI = Harvey Bradshaw Index, MFI–Multidimensional Fatigue Inventory, SFQ = Short Fatigue Questionnaire, SSCAI = Simple Clinical Colitis Activity Index, TS = transferrin saturation.

### Assessment of bias

Bias was assessed using the Newcastle-Ottawa scale modified for cross-sectional studies. The questions were modified for use in this review; see the S2 File for further details. The results are shown in S1 Table for each assessment component and the total score. All studies were categorised as being at medium or high risk of bias. The studies with a high risk of bias had poor comparability for reasons such as not accounting for confounding factors. Nearly all the studies synthesising the primary results were at medium risk of bias.

### Main results synthesis

From the 12 studies assessed, seven studies with 1425 individuals were deemed eligible for inclusion in the main results synthesis. The studies excluded from the man synthesis did not categorise patients as iron deficient and used other analysis methods, such as the correlation between iron status parameters and fatigue. The main results synthesis was sub-grouped as previously described. Study findings are presented in the synthesis Table 3 for IDWA and Table 4 for iron deficiency with any haemoglobin level.

**Table 3:** Main result synthesis for exposure IDWA.

Study	Population	Number	Primary aim of study	Disease activity and inclusion	Exposure: ID or IDWA	Definition of ID and anaemia	NOS Bias	Fatigue PROMs	Results	ID associated with fatigue
Goldenberg *et al*, 2012 [22]	Adult IBD: >18 years	280 CD: 143 UC: 137 Anaemia: 30 Non-anaemic: 230	Investigate the relationship between IDWA and fatigue.	Active and remission. Active disease present in 45.7% (128/280)	IDWA IDWA: 39 Non-IDWA: 191	Anaemia: Hb <140g/L in males, <120g/L in females. ID: ferritin < 20ug/L or sTfR > 28mg/L	6	MFI: 5 domains and total score	High fatigue in 49% of IDWA v 45% in non-ID, (p = 0.73). No significant difference in the 5 MFI domain scores in ID v non-ID.	No
Gonzalez Alayon *et al*, 2017 [38]	Adult IBD: age 18–70 years	127 CD: 78 UC: 49 16 anaemic patients removed from analysis.	Prevalence of IDWA in outpatients and impact of IDWA on HrQOL and fatigue.	Any disease activity. Inflammation present in 21.3%	IDWA 111 non-anaemic: IDWA: 47 Non-IDWA: 64	Anaemia: Hb <130g/L in males, <120g/L in females. ID: ferritin <30μg/L or ferritin <100μg/L in inflammation and TS <16%	7	FACIT-F: lower score indicates fatigue ≤ 30 = extreme fatigue	Lower FACIT-F scores in IDWA than non-ID 37.9 v 42.2 (p = 0.037). Greater prevalence of extreme fatigue in IDWA than non-ID, 64.7% v 35.3% (p = 0.069)	Yes
Herrera-deguise *et al*, 2016 [19]	Adult IBD	104 CD: 58 UC: 46	Evaluate influence of IDWA on HrQOL and fatigue.	Remission only.	IDWA IDWA: 45 Non-IDWA: 59	Anaemia: Hb <13g/dL in males and <12g/dL in females. ID: ferritin <30μg/L and TS < 16%.	6	Fatigue Impact Scale: score from 0–32, higher score more fatigue	IDWA had higher score than non-ID, 8 v 3, (p<0.05), indicating greater fatigue.	Yes

**Table 4:** Main results synthesis for exposure ID in any level of haemoglobin.

Study	Population	Number	Primary aim of study	Disease activity and inclusion	Exposure: ID or IDWA	Definition of ID and anaemia	NOS Bias	Fatigue PROMs	Results	ID associated with fatigue
Jonefjall *et al*, 2018 [20]	Adult UC	288 UC only	Prevalence of high fatigue and risk factors for fatigue in UC in remission and active disease	Active (n = 155) and remission (n = 133)	ID ID: 51 Non-ID: 208 29 not classified (missing data) Anaemia = 13	ID: Ferritin <30μg/L or in active disease ferritin 30–100μg/L with either 1) TS <16%/ or raised sTfR or 2) TS <20% and raised CRP.	7	MFI: 5 domains and total score. Fatigue ≥13 in general fatigue domain	Higher prevalence of ID in high fatigued, 32% v mild/no fatigue, 19% (p <0.001). Multivariate logistic regression for ID as risk factor for fatigue: OR 2.5 (CI 1.2–5.1).	Yes
Konig *et al*, 2020 [24]	Adult IBD: age >18 years	98 UC: 28 CD: 70	Determine the influence of ID, anaemia and inflammation on fatigue, anxiety, depression	Any disease activity	ID ID: 34, Non-ID: 64	ID = ferritin <30μg/L or ferritin <100μg/L and TS <20% if CRP >0.5mg/dL	7	Piper Fatigue Scale	42% of patients with ID were fatigued v 23% without ID (p = 0.06). Multiple logistic regression for ID as risk factor for fatigue: OR 2.1 (CI 0.8–5.8), p = 0.13	Tendency to fatigue but not statistically significant
Aluzuite *et al*, 2019 [39]	Adult IBD: >18 years	113 UC: 43 CD: 79	Prevalence of fatigue and associated variables.	Active (n = 70) Remission (n = 43)	ID ID: 54 Non-ID: 59	ID: ferritin < 30μg/L or <100μg/L in inflammation.	6	1.MFI 2.BFI	No data provided. No association between presence of ID and fatigue measures reported.	No
Bager *et al*, 2012	Adult IBD	425 UC: 174 CD: 251	Prevalence of fatigue and determinates of fatigue	Any disease activity.	ID ID: 146 Non ID: 279	ID: ferritin < 30μg/L or <100μg/L in inflammation	4	MFI-20: 5 dimensions of fatigue	No data provided. No difference in fatigue scores in ID v non-ID reported.	No

### Description of results

In the IDWA synthesis, three studies directly compared fatigue scores in IDWA to those with normal haemoglobin and iron status. In two studies, Gonzalez et al. [38] and Herrera-Deguise et al. [19] showed patients with IDWA in IBD had significantly worse fatigue than non-iron deficient patients. In these studies, the average fatigue scores in both groups of iron status were not in the fatigued range, according to the PROM fatigue measurement used. Both studies included mixed groups of Crohn’s disease (CD) and ulcerative colitis (UC). Gonzalez et al. also showed that the prevalence of severe fatigue was almost twice as high in iron-deficient patients, though this was just outside statistical significance: the third study, Goldenberg et al. [22], showed no significant difference in fatigue in different fatigue domains or prevalence of fatigue between those who were iron deficient and those who were not. More patients with active disease were in the Goldenberg study than the others and a lower ferritin cut-off was used to define iron deficiency.

In the synthesis for ID with any haemoglobin level, two studies, Bager et al. [18] and Aluzaite et al., [23] reported no association between iron deficiency and fatigue. However, they did not present their results in these reports. They were included to avoid selection bias by excluding negative studies. The other two studies, Konig et al. [24] and Jonefjall et al. [20] presented the prevalence of iron deficiency with fatigue. Jonefjall et al. was the only study which showed a higher prevalence of fatigue in patients with ID and any level of haemoglobin, which was statistically significant. This study contained only UC patients and had a few anaemic patients, 5% of the total. All four of these studies included patients with any level of disease activity.

### Certainty of the evidence

Using the GRADE for systematic reviews without a single effect measure [40], see Table 5.

**Table 5:** GRADE assessment for certainty of evidence.

GRADE domain	Judgement	Concerns about certainty domains
Methodological limitations of the studies	The risk of bias scores were medium or low for studies included. Many of the studies did not plan to be adequately powered to detect differences in fatigue levels and were primarily interested in a different outcome, such as quality of life. The populations varied by types of IBD and disease activity, these were often not controlled for.	**Serious**
Indirectness:	There was a direct comparison between iron deficiency and non-iron deficient patients in the studies in the main results synthesis. There was variability in outcomes measures used which may have measured differing aspects of fatigue.	**Borderline**
Imprecision:	There were 511 patients included in the IDWA results synthesis and 895 in the results synthesis for any haemoglobin level. There were small numbers in some individual studies which may have been underpowered.	**Borderline**
Inconsistency:	There was inconsistency in the results in both subgroups. In the IDWA synthesis there was an increase in fatigue levels in two studies associated with IDWA [19, 38]. The difference in fatigue was small in both studies and average fatigue score in the IDWA groups did not indicate severe fatigue. Goldenberg et al [22] showed no significant difference in fatigue levels across different domains of fatigue and overall. In the synthesis for any haemoglobin level, one study of UC patients only showed a difference in fatigue, compared to the other studies which were CD and UC. The two studies which provided results data used proportion of patients with fatigue in ID and non-ID. The statistical analysis used the studies with no results data is not clear.	**Serious**
Publication bias:	Not strongly suspected as both negative and positive trials were included.	**Not suspected**

Overall certainty of the evidence is low due to the risk of bias and inconsistency of results.

### Limitations of synthesis

The limitations of this synthesis are several. Only a few studies were eligible to be included in the main results synthesis due to the lack of definite exposure to iron deficiency in many studies. Different PROMs were used to measure the outcome of fatigue between studies which may have assessed variable qualities of fatigue, such as mental or physical, and therefore are not directly comparable. The cut-off scores indicating fatigue vary between PROMs, so interpretation must be guided by this. Due to this, it was not possible to create a standardised effect measure of iron deficiency. The prevalence data from the synthesis, including any haemoglobin level, could be converted to odds ratios and combined in a meta-analysis. Still, this would have limitations due to the Jonefjall [20] study containing patients with UC only and no data available from the other two studies. There was considerable heterogeneity between studies for factors that may influence fatigue levels, such as differing disease activity levels, which limits the validity of combining the results overall. In the synthesis, including any haemoglobin level, there were different numbers of anaemic patients in the individual studies, which may have contributed to the fatigue levels. All but one of the studies included mixed populations of ulcerative colitis and Crohn’s disease patients. Fatigue is more severe in Crohn’s disease [41], so investigating these diseases separately would be preferable.

### Studies not included in the synthesis of the main results

Studies that categorised patients by fatigue scores, then determined iron status at different levels of fatigue, or correlation analyses to investigate the relationship between fatigue and iron status were excluded from synthesising the main results. The results are described here and can be found in S2 Table. Three studies (Chavarria et al., Banovic et al., Villoria et al. [33, 34, 37]) categorised patients according to different fatigue levels and presented the mean ferritin levels in each group. The mean ferritin levels in the groups of non-fatigued and fatigued patients were all in the normal range in these studies. In one study, there were lower mean ferritin levels in the severe fatigue group (Villoria et al.) of 85μg/L v 134μg/L in mild fatigue and 131μg/L in no fatigue, though this was not statistically significant. The Chavarria et al. study showed similar ferritin levels in fatigue and non-fatigue, 91.3μg/L v 90.8μg/L. Banovic et al. showed lower ferritin levels in the less fatigued group vs the high fatigued group, 59.94μg/L v 69.25μg/L, respectively. This study included patients in remission only, whereas Chavarria et al. and Villoria et al. included different disease activity levels. According to guidelines, the cut-off for ferritin to diagnose iron deficiency in inflammation changes to ferritin <100μg/L [41]. No information was provided regarding the analysis of ferritin results in patients with inflammation and if cut-offs were adjusted. This may bias the results towards higher ferritin levels in patients with inflammation. The lower ferritin levels observed in the Banovic et al. [33] study may be due to the study population being in remission and ferritin levels being lower in the absence of inflammation.

Two other studies analysed the relationship of iron parameters to fatigue levels. Grimstad et al. [35] analysed ferritin levels and fatigue PROMs by regression analysis in patients newly diagnosed with IBD. It was also not clear if ferritin cut-offs were adjusted according to the presence of inflammation. There was an association of ferritin levels with fatigue score when adjusted for age and gender but not in multiple linear regression. Truyens et al. [36] conducted a univariable linear regression of fatigue and iron deficiency using transferrin saturation, followed by multiple regression analysis, which showed increased fatigue was significantly related to lower transferrin saturation levels.

## Discussion

This systematic review aimed to determine from existing literature if iron deficiency in patients with IBD is associated with fatigue. The studies showed varied results for the association of fatigue and iron deficiency. This may be explained by the heterogeneity of studies, particularly the clinical diversity of the participants included and outcome measures used, the study design and statistical methods. Active inflammation in IBD contributes to fatigue, so this may have been a confounder of studies with mixed disease activity. The two studies in patients with IDWA showing worse fatigue levels are associated with iron deficiency, and they were in populations predominantly in remission. However, in the other study by Goldenberg et al. in IDWA, which found no association, approximately half the patients had active disease, and the plasma ferritin cut-off value was not adjusted in inflammation. Soluble transferrin receptor levels were used alongside ferritin to account for this. This marker is intended for anaemia, though it has been used in IDWA for some time. Goldenberg et al. also used a lower ferritin cut-off to define iron deficiency, resulting in some patients who would have been classified as iron deficient in other studies being in the normal iron status group. Overall, this indicates there may be an association between excessive fatigue and IDWA in patients in remission. In the two studies demonstrating this, the average fatigue PROM score in both non-iron deficient and iron deficient groups was not in the range used to define severe fatigue. The difference in FACIT-F score between the iron deficient and non-iron deficient was only 4.3 points [38].

In contrast, a 7–10 point improvement for CD and a 4–9 point improvement for UC may represent meaningful improvements [42]. The relationship between IDWA and worse fatigue is statistically significant. Still, the effect size appears small, so it may not be clinically important.

In synthesising the results for any haemoglobin level, three studies showed that iron deficiency was unrelated to fatigue in cohorts of mixed Ulcerative Colitis and Crohn’s disease patients. The lack of data on the results of two of these studies made it challenging to interpret this further. Jonefjall et al. showed a greater prevalence of iron deficiency in fatigued ulcerative colitis patients. It is unclear if limiting to a UC population was a factor in these results. This study included a low number of anaemic patients, limiting the effect of including anaemic patients on fatigue levels. Analysis of results for fatigue in anaemic patients may have been more revealing in classifying patients as having IDA or anaemia of inflammation than analysing anaemia altogether.

The studies included in both parts of the main synthesis did not control for other factors influencing fatigue, such as mental health status or poor sleep. It is challenging to ascertain the exact contribution of iron deficiency to overall fatigue in IBD. The measurements of fatigue used in these studies are subjective, meaning that the nature of PROMs means self-perceived fatigue may vary between individuals, which may also be a limiting factor in reviewing the evidence.

There were few studies where iron replacement was administered as an intervention to investigate changes in fatigue levels, and these were not included in the systematic review. This may limit the application of the findings to clinical practice in treating fatigue. ECCO guidelines highlight that fatigue can occur in IBD independent of anaemia or iron deficiency [41] and recommend treating anaemia to improve quality of life. Treatment of iron deficiency without anaemia is also advised to improve numerous symptoms, including fatigue [41] based on evidence mostly from non-IBD populations. The findings of this review support this recommendation, especially in patients in remission, though further work is required to fully understand the relationship between iron deficiency and fatigue. Future study design should control for other factors impacting fatigue, such as by studying non-anaemic patients in remission only and in separate cohorts of UC and Crohn’s disease patients. Objective measures of fatigue should be used for outcome measures. Trials of iron replacement in patients with fatigue and iron deficiency could be used to investigate if there was a causative relationship between iron deficiency and fatigue in IBD and inform management of fatigue.

## Conclusions

Considerable variation in the cohort under investigation, especially differences in IBD phenotype and disease activity used to investigate the relationship between iron deficiency and fatigue were found in this systematic review. There were substantial differences in methods used to define iron deficiency, outcome measures to assess fatigue and statistical analysis approach. There is some evidence that IDWA is associated with worsening fatigue, particularly in patients in clinical remission, though the degree of increased fatigue may not be clinically important. Further studies are required to support this conclusion. Iron deficiency may play a role in fatigue but does not explain the full extent of fatigue in patients with IBD. The exact contribution of iron deficiency to fatigue in IBD requires further investigation, but future studies should provide detailed reporting of the population, including subgroup analysis for diagnosis (UC or Crohn’s Disease) and disease activity, and measures of iron status should be kept in keeping with clinical guidelines. Validated outcome measures for fatigue in patients with IBD, including objective assessments, should be used. Other causes of fatigue need to be considered and controlled for in the study design.

## Supporting information

S1 Checklist SWIM checklist. (DOCX)

S2 Checklist PRISMA checklist. (DOCX)

S1 File Search strategies. (DOCX)

S2 File Newcastle-Ottawa score adapted. (DOCX)

S3 File Supporting information. (CSV)

S1 Table Assessment of bias results. (DOCX)

S2 Table Data extraction table. (DOCX)
