# Supplementary material for: Iron deficiency and fatigue in inflammatory bowel disease: A systematic review
Source: PLoS One. 2025 Jan 13;20(1):e0304293. doi: 10.1371/journal.pone.0304293 (PMC11730394; doi:10.1371/journal.pone.0304293)
Supplement: S1 File — (DOCX) [file pone.0304293.s003.docx]

# Supplementary material 1: Search strategies

## Medline via Ovid

Date searched: 25/10/2022

Search results: 48

Ovid MEDLINE(R) and Epub Ahead of Print, In-Process, In-Data-Review & Other Non-Indexed Citations, Daily and Versions <1946 to September 14, 2022>

1 inflammatory bowel diseases/ or exp colitis, ulcerative/ or exp crohn disease/ or exp proctitis/

2 "inflammatory bowel disease$".mp.

3 "crohn$ disease".mp.

4 "ulcerative colitis".mp.

5 1 or 2 or 3 or 4

6 exp fatigue/ or exp mental fatigue/

7 exp Muscle Fatigue/

8 fatigue.mp.

9 tired$.mp.

10 letharg$.mp.

11 exp iron deficiencies/ or exp anemia, iron-deficiency/

12 exp iron compounds/ or exp ferric compounds/ or exp ferrous compounds/ or exp iron, dietary/

13 exp ferritins/ or exp transferrins/

14 ferritin.mp.

15 "transferrin sat$".mp.

16 "iron deficienc$".mp.

17 6 or 7 or 8 or 9 or 10

18 11 or 12 or 13 or 14 or 15 or 16

19 5 and 17 and 18

No limits set

# Embase via Ovid

Date searched: 22/11/2022 Results = 233

Database: Embase Classic+Embase <1947 to 2022 November 21> Search Strategy:

--------------------------------------------------------------------------------

1 inflammatory bowel disease/ or digestive system inflammation/ or crohn disease/ or experimental inflammatory bowel disease/ or ulcerative colitis/ (191677)

2 "inflammatory bowel disease*".ti,ad,kw. (59208)

3 "Crohn* disease".ti,ab,kw. (92863)

4 "ulcerative colitis".ti,ab,kw. (80727)

5 1 or 2 or 3 or 4 (212655)

6 exp mental fatigue/ or exp fatigue/ (296658)

7 exp muscle fatigue/ (14372)

8 fatigue.mp. [mp=title, abstract, heading word, drug trade name, original title, device manufacturer, drug manufacturer, device trade name, keyword heading word, floating subheading word, candidate term word] (332571)

9 tired*.mp. [mp=title, abstract, heading word, drug trade name, original title, device manufacturer, drug manufacturer, device trade name, keyword heading word, floating subheading word, candidate term word] (12068)

10 letharg*.mp. [mp=title, abstract, heading word, drug trade name, original title, device manufacturer, drug manufacturer, device trade name, keyword heading word, floating subheading word, candidate term word] (32183)

11 6 or 7 or 8 or 9 or 10 (377384)

12 exp iron blood level/ (15079)

13 exp iron therapy/ or exp diet therapy/ or exp supplementation/ (550308)

14 exp iron deficiency/ or exp iron deficiency anemia/ or exp iron depletion/ (51958)

15 iron deficiency/ or exp hypoferremia/ (20626)

16 exp ferritin blood level/ or exp ferritin/ (66869)

17 exp transferrin blood level/ or exp transferrin/ or exp transferrin saturation/ (37050)

18 ferritin.mp. [mp=title, abstract, heading word, drug trade name, original title, device manufacturer, drug manufacturer, device trade name, keyword heading word, floating subheading word, candidate term word] (74501)

19 "transferrin sat*".mp. [mp=title, abstract, heading word, drug trade name, original title, device manufacturer, drug manufacturer, device trade name, keyword heading word, floating subheading word, candidate term word] (7830)

20 "iron deficienc*".mp. [mp=title, abstract, heading word, drug trade name, original title, device manufacturer, drug manufacturer, device trade name, keyword heading word, floating subheading word, candidate term word] (57752)

21 12 or 13 or 14 or 15 or 16 or 17 or 18 or 19 or 20 (682553)

22 5 and 11 and 21 (440)

23 limit 22 to human (427)

24 limit 23 to (adult <18 to 64 years> or aged <65+ years>) (233)

## CINAHL

Date searched: 23/11/2022

| **Search ID#** | **Search Terms** | **Search Options** | **Last Run Via** | **Results** |
| --- | --- | --- | --- | --- |
| S1 | (MH "Inflammatory Bowel Diseases+") | Expanders - Apply equivalent subjects Search modes - Find all my search terms | Interface - EBSCOhost Research Databases Search Screen - Advanced Search Database - CINAHL Plus with Full Text | 18,882 |
| S2 | (MH "Colitis, Ulcerative") | Expanders - Apply equivalent subjects Search modes - Find all my search terms | Interface - EBSCOhost Research Databases Search Screen - Advanced Search Database - CINAHL Plus with Full Text | 6,295 |
| S3 | (MH "Crohn Disease") | Expanders - Apply equivalent subjects Search modes - Find all my search terms | Interface - EBSCOhost Research Databases Search Screen - Advanced Search Database - CINAHL Plus with Full Text | 8,027 |
| S4 | "inflammatory bowel diseas*" | Expanders - Apply equivalent subjects Search modes - Find all my search terms | Interface - EBSCOhost Research Databases Search Screen - Advanced Search Database - CINAHL Plus with Full Text | 13,508 |
| S5 | "crohn* disease" | Expanders - Apply equivalent subjects Search modes - Find all my search terms | Interface - EBSCOhost Research Databases Search Screen - Advanced Search Database - CINAHL Plus with Full Text | 10,922 |
| S6 | "ulcerative colitis" | Expanders - Apply equivalent subjects Search modes - Find all my search terms | Interface - EBSCOhost Research Databases Search Screen - Advanced Search Database - CINAHL Plus with Full Text | 7,605 |
| S7 | S1 OR S2 OR S3 OR S4 OR S5 OR S6 | Expanders - Apply equivalent subjects Search modes - Find all my search terms | Interface - EBSCOhost Research Databases Search Screen - Advanced Search Database - CINAHL Plus with Full Text | 24,330 |
| S8 | (MH "Fatigue+") OR (MH "Mental Fatigue+") OR (MH "Muscle Fatigue") | Expanders - Apply equivalent subjects Search modes - Find all my search terms | Interface - EBSCOhost Research Databases Search Screen - Advanced Search Database - CINAHL Plus with Full Text | 30,820 |
| S9 | "fatigue" | Expanders - Apply equivalent subjects Search modes - Find all my search terms | Interface - EBSCOhost Research Databases Search Screen - Advanced Search Database - CINAHL Plus with Full Text | 54,618 |
| S10 | "tired*" | Expanders - Apply equivalent subjects Search modes - Find all my search terms | Interface - EBSCOhost Research Databases Search Screen - Advanced Search Database - CINAHL Plus with Full Text | 3,237 |
| S11 | "letharg*" | Expanders - Apply equivalent subjects Search modes - Find all my search terms | Interface - EBSCOhost Research Databases Search Screen - Advanced Search Database - CINAHL Plus with Full Text | 1,484 |
| S12 | S8 OR S9 OR S10 OR S11 | Expanders - Apply equivalent subjects Search modes - Find all my search terms | Interface - EBSCOhost Research Databases Search Screen - Advanced Search Database - CINAHL Plus with Full Text | 58,081 |
| S13 | (MH "Iron Deficiencies") OR (MH "Anemia, Iron Deficiency") | Expanders - Apply equivalent subjects Search modes - Find all my search terms | Interface - EBSCOhost Research Databases Search Screen - Advanced Search Database - CINAHL Plus with Full Text | 4,933 |
| S14 | (MH "Iron Compounds+") | Expanders - Apply equivalent subjects Search modes - Find all my search terms | Interface - EBSCOhost Research Databases Search Screen - Advanced Search Database - CINAHL Plus with Full Text | 5,590 |
| S15 | (MH "Ferritin") | Expanders - Apply equivalent subjects Search modes - Find all my search terms | Interface - EBSCOhost Research Databases Search Screen - Advanced Search Database - CINAHL Plus with Full Text | 3,781 |
| S16 | (MH "Transferrin") | Expanders - Apply equivalent subjects Search modes - Find all my search terms | Interface - EBSCOhost Research Databases Search Screen - Advanced Search Database - CINAHL Plus with Full Text | 1,342 |
| S17 | ferritin | Expanders - Apply equivalent subjects Search modes - Find all my search terms | Interface - EBSCOhost Research Databases Search Screen - Advanced Search Database - CINAHL Plus with Full Text | 6,287 |
| S18 | "transferrin sat*" | Expanders - Apply equivalent subjects Search modes - Find all my search terms | Interface - EBSCOhost Research Databases Search Screen - Advanced Search Database - CINAHL Plus with Full Text | 808 |
| S19 | "iron deficienc*" | Expanders - Apply equivalent subjects Search modes - Find all my search terms | Interface - EBSCOhost Research Databases Search Screen - Advanced Search Database - CINAHL Plus with Full Text | 7,886 |
| S20 | S13 OR S14 OR S15 OR S16 OR S17 OR S18 OR S19 | Expanders - Apply equivalent subjects Search modes - Find all my search terms | Interface - EBSCOhost Research Databases Search Screen - Advanced Search Database - CINAHL Plus with Full Text | 16,785 |
| S21 | S7 AND S12 AND S20 | Expanders - Apply equivalent subjects Search modes - Find all my search terms | Interface - EBSCOhost Research Databases Search Screen - Advanced Search Database - CINAHL Plus with Full Text | 15 |

### Web of Science

Date searched: 22/05/2023

Search strategy: "inflammatory bowel disease*" OR "Crohn* disease" OR "ulcerative colitis" (Topic) AND "iron deficienc*" OR "ferritin" OR "transferrin sat*" (Topic) AND "fatigu*" OR "tired" (Topic)
