# Supplementary material for: Iron deficiency and fatigue in inflammatory bowel disease: A systematic review
Source: PLoS One. 2025 Jan 13;20(1):e0304293. doi: 10.1371/journal.pone.0304293 (PMC11730394; doi:10.1371/journal.pone.0304293)
Supplement: S2 File — (DOCX) [file pone.0304293.s004.docx]

# Newcastle-Ottawa Scale adapted for cross-sectional studies

This scale has been adapted from the Newcastle-Ottawa Quality Assessment Scale for cohort studies, as previously done by Herzog et al(Herzog et al., 2013).

**Selection: (Maximum 5 stars)**

1. Representativeness of the sample:
   1. Truly representative of the average in the target population. * (all subjects or random sampling)
   2. Somewhat representative of the average in the target group. * (non-random sampling)
   3. Selected group of users.
   4. No description of the sampling strategy.
2. Sample size:
   1. Justified and satisfactory (including sample size calculation). *
   2. Not justified.
   3. No information provided
3. Non-respondents:

a. Comparability between respondents and non-respondents characteristics is established, and the response rate is satisfactory. *

b. The response rate is unsatisfactory, or the comparability between respondents and non-respondents is unsatisfactory.

c. No description of the response rate or the characteristics of the responders and the non-responders.

1. Ascertainment of the exposure (risk factor):
   1. Blood test results provided with definitions for anaemia and iron deficiency stated, including adjusting ferritin cut-off for iron deficiency adjusted in the presence of inflammation. **
   2. Blood test results provided with definitions stated for iron deficiency and anaemia. *
   3. No results for blood tests provided.

**Comparability:** (Maximum 2 stars)

1. Comparability of subjects in different outcome groups on the basis of design or analysis. Confounding factors controlled.
   1. Data/ results adjusted for relevant predictors/risk factors/confounders e.g. age, sex, disease activity in multivariate analysis**
   2. One aspect controlled such as disease activity or anaemia *
   3. Data/results not adjusted for all relevant confounders/risk factors/information not provided.

**Outcome:** (Maximum 3 stars)

1. Assessment of outcome:
   1. Using objective validated methods. **
   2. Self-reported outcome. *
   3. No description.
2. Statistical test:
   1. Statistical test used to analyse the data clearly described, appropriate and measures of association presented including confidence intervals and probability level (p value). *
   2. Statistical test not appropriate, not described or incomplete. No results/description provided.

Cross-sectional Studies:

Good Studies: 8- 10 points

Satisfactory Studies: 5-7 points

Unsatisfactory Studies: 0 to 4 points

HERZOG, R., ÁLVAREZ-PASQUIN, M. J., DÍAZ, C., DEL BARRIO, J. L., ESTRADA, J. M. & GIL, Á. 2013. Are healthcare workers' intentions to vaccinate related to their knowledge, beliefs and attitudes? A systematic review. *BMC Public Health,* 13**,** 154.
