# Supplementary material for: Iron deficiency and fatigue in inflammatory bowel disease: A systematic review
Source: PLoS One. 2025 Jan 13;20(1):e0304293. doi: 10.1371/journal.pone.0304293 (PMC11730394; doi:10.1371/journal.pone.0304293)
Supplement: S1 Table — (DOCX) [file pone.0304293.s006.docx]

**Table 3: Assessment of bias results**

| Author | Selection | Comparability | Outcome | Total |
| --- | --- | --- | --- | --- |
| Aluzaite et al[1] | *** | ** | * | 6 |
| Bager et al[2] | *** |  | * | 4 |
| Banovic et al[3] | ** |  | ** | 4 |
| Chavarria et al[4] | ** |  | ** | 4 |
| Goldenberg et al[5] | *** | * | ** | 6 |
| Gonzalez et al[6] | **** | * | ** | 7 |
| Grimstad et al[7] | ** | ** | ** | 6 |
| Herrera-Deguise et al[8] | *** | * | ** | 6 |
| Jonefjall et al[9] | *** | ** | ** | 7 |
| Konig et al[10] | *** | ** | ** | 7 |
| Truyens et al[11] | *** | ** | ** | 7 |
| Villoria et al[12] | *** |  | ** | 5 |

Interpretation of total results score: 8-10 = low risk of bias, 5-7 = medium risk of bias, 4 and under = high risk of bias.

References:

1. Aluzaite K, Al-Mandhari R, Osborne H, Ho C, Williams M, Sullivan M-M, et al. Detailed Multi-Dimensional Assessment of Fatigue in Inflammatory Bowel Disease. Inflammatory intestinal diseases. 2019;3(4):192-201.

2. Bager P. Fatigue and acute/chronic anaemia. Danish medical journal. 2014;61(4):B4824.

3. Banovic I, Gilibert D, Jebrane A, Cosnes J. Personality and fatigue perception in a sample of IBD outpatients in remission: a preliminary study. J Crohns Colitis. 2012;6(5):571-7.

4. Chavarría C, Casanova MJ, Chaparro M, Barreiro-de Acosta M, Ezquiaga E, Bujanda L, et al. Prevalence and Factors Associated With Fatigue in Patients With Inflammatory Bowel Disease: A Multicentre Study. J Crohns Colitis. 2019;13(8):996-1002.

5. Goldenberg BA, Graff LA, Clara I, Zarychanski R, Walker JR, Carr R, et al. Is iron deficiency in the absence of anemia associated with fatigue in inflammatory bowel disease? American Journal of Gastroenterology. 2013;108(9):1392-7.

6. Gonzalez Alayon C, Pedrajas Crespo C, Marin Pedrosa S, Benitez JM, Iglesias Flores E, Salgueiro Rodriguez I, et al. Prevalence of iron deficiency without anaemia in inflammatory bowel disease and impact on health-related quality of life. Prevalencia de deficit de hierro sin anemia en la enfermedad inflamatoria intestinal y su impacto en la calidad de vida. 2018;41(1):22-9.

7. Grimstad T, Norheim KB, Isaksen K, Leitao K, Hetta AK, Carlsen A, et al. Fatigue in Newly Diagnosed Inflammatory Bowel Disease. JOURNAL OF CROHNS & COLITIS. 2015;9(9):725-30.

8. Herrera-Deguise C, Casellas F, Robles V, Navarro E, Borruel N. Iron deficiency in the absence of anemia impairs the perception of health-related quality of life of patients with inflammatory bowel disease. Inflammatory Bowel Diseases. 2016;22(6):1450-5.

9. Jonefjall B, Simren M, Lasson A, Ohman L, Strid H. Psychological distress, iron deficiency, active disease and female gender are independent risk factors for fatigue in patients with ulcerative colitis. United European gastroenterology journal. 2018;6(1):148-58.

10. König P, Jimenez K, Saletu-Zyhlarz G, Mittlböck M, Gasche C. Iron deficiency, depression, and fatigue in inflammatory bowel diseases. Zeitschrift fur Gastroenterologie. 2020;58(12):1191-200.

11. Truyens M, De Ruyck E, Gonzales GB, Bos S, Laukens D, De Vos M. Prevalence of Fatigue and Unrecognized Depression in Patients with Inflammatory Bowel Disease in Remission under Immunosuppressants and Biologicals. J Clin Med. 2021;10(18).

12. Villoria A, García V, Dosal A, Moreno L, Montserrat A, Figuerola A, et al. Fatigue in out-patients with inflammatory bowel disease: Prevalence and predictive factors. PLoS One. 2017;12(7):e0181435.
