# Supplementary material for: Iron deficiency and fatigue in inflammatory bowel disease: A systematic review
Source: PLoS One. 2025 Jan 13;20(1):e0304293. doi: 10.1371/journal.pone.0304293 (PMC11730394; doi:10.1371/journal.pone.0304293)
Supplement: S2 Table — (DOCX) [file pone.0304293.s007.docx]

| **Study** | **Population** | **Number** | **Type of study** | **Primary aim of study** | **Disease activity and inclusion** | **Methods** | **Exposure: ID or IDWA** | **Definition of ID and anaemia** | **Fatigue PROMs** | **Results** | **ID associated with fatigue** |
| --- | --- | --- | --- | --- | --- | --- | --- | --- | --- | --- | --- |
| Banovic et al, 2011 (1) | Adult IBD | 81  CD: 59  UC: 22 | Prospective, observational, single centre, out-patients | Determine the relationship between personality and perception of IBD related fatigue in remission. | Remission only. CD HBI <5, UC Lichtiger Index < 7. Patients selected for reporting fatigue | Measuring plasma ferritin in high and low fatigue categories. | Not applicable | Ferritin and CRP measured - no further info given | MFI global score:  high fatigue = MFI > 43  low fatigue = MFI ≤43 | Mean plasma ferritin in low fatigue: 59.94 (+/-56.44)mg/L v high fatigue: 69.25 (+/-57.05)mg/L p> 0.05. | No |
| Chavarria et al, 2019 (2) | Adult IBD > 18 years | 544  CD: 331  UC: 211 | Multicentre, observational, prospective study | Determine the prevalence of fatigue in patients with IBD, identify factors associated with fatigue and severity, assess the impact of fatigue on QOL and relationship disorders. | All disease activity. Active disease in CD: HBI > 4, in UC partial Mayo ≥ 2. | Ferritin levels in high and low fatigue categories in univariate analysis. Multivariate analysis of association between different variables and fatigue severity. | Not applicable | Anaemia: Hb <13g/dL in males and <12g/dL in females. ID = ferritin < 30ng/ml without inflammation and < 100ng/ml with inflammation. | Fatigue Severity Scale: fatigue = score ≥5 used in analysis categories. Fatigue Impact Scale: no cut-off given. | Ferritin in fatigue v non-fatigue is 91.3 (70.6 - 114.5)ng/ml v 90.8 (79.6 - 103.2)ng/ml. No statistical difference in Hb and ferritin levels with or without fatigue. | No |
| Grimstad et al, 2015 (3) | Adult IBD > 16 | 81  CD: 21  UC: 60 | Single centre, prospective, observational | Describe the prevalence and degree of fatigue in a cohort of newly diagnosed and untreated UC and CD patients compared to matched healthy controls. | Newly diagnosed IBD. Inflammation = CRP ≥5 ug/ml and calprotectin ≥ 50mg/kg | IBD patients v healthy controls. Associations with fatigue in IBD patients regression analyses. 1. Age and sex-adjusted regression analysis and 2. Fully adjusted model | Not applicable | ID = ferritin < 30µg/L if CRP < 5mg/L and ferritin < 100µg/L if CRP ≥ 5mg/L. No information for haemoglobin. | fVS: fatigue ≥50 and FSS: fatigue ≥4 | fVAS associated with ferritin in multiple regression adjusted for age and gender but not in fully adjusted model. | No in fully adjusted model. |
| Truyens et al, 2021 (4) | Adult IBD | 157  CD: 123  UC: 34 | Prospective, observational, single centre, tertiary centre | Determine the prevalence of fatigue and its correlation with depression in an IBD population in remission. | Remission determined by HBI ≤3 or clinical Mayo score ≤ 1 and in biochemical remission CRP ≤ 10mg/L. Excluded flare in last 6 months, anaemia, depression. | Univariate linear regression of fatigue and iron deficiency (using transferrin saturations), then multivariate regression. | Not applicable | No definition of iron deficiency given. Measured transferrin saturations and ferritin. Anaemia: Hb < 11.8g/dL. | SFQ: fatigue ≥18  fVAS: fatigue = ≥5 | Higher transferrin saturation (TS) associated with lower fatigue in multivariate regression. Statistically significant. Univariate linear regression OR for fatigue using TS: -0.07 [-0.16-0.80] Adj p value = 0.269. Multivariate OR = -0.11[-0.22--0.01] p = 0.037 | Yes in multivariate analysis |
| Villoria et al, 2017(5) | Adult IBD ≥ 18 years | 177  CD: 127 UC: 50 | Prospective, observational, outpatients, single centre | Investigate biological factors associated with fatigue in IBD, including pro-inflammatory cytokines and micronutrients | Excluded if flare in last 3 months. HBI ≤ 5 in 90% and ≤ 7 in 98% CD, Mayo ≤2 in 78%, ≤4 in 93% UC. | Ferritin levels in 3 categories of fatigue score by FACIT-F score: > 40, 20-40, <20 | Not applicable | No definition of iron deficiency given. Measured transferrin saturations, ferritin and iron. | FACIT-F | Ferritin levels in: No fatigue (FS>40) = 131+/113ng/ml, Mild fatigue (FS 20-40) = 134 +/-137ng/ml, Severe fatigue (FS<20) = 85 +/-78ng/ml. p = 0.16 | No |
| Goldenberg et al, 2012 (6) | Adult IBD > 18 years | 280  CD: 143  UC: 137  230 not anaemic | Prospective, observational, cross-sectional from Manitoba cohort | To investigate the relationship between iron deficiency and fatigue in IBD in the absence of anaemia. | Active and remission. 128/280 (46%) have active disease, defined as HBI or Powell Tuck ≥ 5 | Comparing mean fatigue scores in iron deficient v non-iron deficient patients, all without anaemia | IDWA  IDWA: 39  Non-IDWA: 191 | IDWA: Anaemia Hb < 140g/dL in males, < 120g/dL in females, ferritin < 20ug/L or STR > 28mg/L | MFI 5 domains. General Fatigue subscale ≥ 13 = significant fatigue | High fatigue in 49% of IDWA v 45% in non-ID, (p = 0.73). No significant difference in the 5 MFI domain scores in ID v non-ID. | No |
| Gonzalez Alayon *et al,* 2017(7) | Adult IBD: age 18 – 70 years | 127  CD: 78  UC: 49  16 anaemic patients removed from analysis. | Prospective, observational, cross-sectional, single centre | Prevalence of IDWA in outpatients and impact of IDWA on HrQOL and fatigue. | Any disease activity. Inflammation present in 21.3%, defined as CRP > 5 with symptoms of active disease or investigations showing inflammation | Comparing fatigue scores in iron deficient v non-iron deficient patients. Prevalence of extreme fatigue FACIT F < 30. | IDWA  111 non-anaemic:  IDWA: 47  Non-IDWA: 64 | Anaemia: Hb <130g/L in males, <120g/L in females. ID: ferritin <30µg/L or ferritin <100µg/L in inflammation and TS <16% | FACIT-F: lower score indicates fatigue  ≤ 30 = extreme fatigue | Lower FACIT-F scores in IDWA than non-ID 37.9 v 42.2 (p = 0.037).  Greater prevalence of extreme fatigue in IDWA than non-ID, 64.7% v 35.3% (p = 0.069) | Yes |
| Herrera-deguise *et al,* 2016(8) | Adult IBD | 104  CD: 58  UC: 46 | Prospective, observational, cross-sectional | Evaluate the influence of IDWA on HrQOL and fatigue in IBD in clinical remission. | Remission only. | Comparing fatigue scores in iron deficient v non-iron deficient patients. | IDWA  IDWA: 45  Non-IDWA: 59 | Anaemia: Hb <13g/dL in males and <12g/dL in females. ID: ferritin <30µg/L and TS < 16%. | Fatigue Impact Scale: score from 0 -32, higher score more fatigue | IDWA had higher score than non-ID, 8 v 3, (p<0.05), indicating greater fatigue. | Yes |
| Jonefjall *et al,* 2018(9) | Adult UC | 288  UC only | Prospective, observational, cross-sectional, outpatients, multicentre | Investigate prevalence of high fatigue and risk factors for fatigue in UC in remission and active disease | Active (n = 155) and remission (n = 133) | Comparing the prevalence of iron deficiency in high fatigue v no fatigue | ID  ID: 51  Non-ID: 208  29 not classified (missing data)  Anaemia =13 | ID: Ferritin <30µg/L or in active disease ferritin 30-100µg/L with either 1) TS <16%/ or raised sTfR or 2) TS <20% and raised CRP | MFI: 5 domains and total score. Fatigue ≥13 in general fatigue domain | Higher prevalence of ID in fatigued, 32% v mild/no fatigue, 19% (p <0.001).  Multivariate logistic regression for ID as risk factor for fatigue: OR 2.5 (CI 1.2 – 5.1). | Yes |
| Konig *et al,* 2020(10) | Adult IBD: age >18 years | 98  UC: 28 CD: 70 | Prospective,  Observational,  single centre, outpatients | Determine the influence of ID, anaemia and inflammation on fatigue, anxiety, depression | Any disease activity | Comparing the prevalence of patients with fatigue in those who have ID v non-ID. | ID  ID: 34,  Non-ID: 64 | ID = ferritin <30µg/L or ferritin <100µg/L and TS <20% if CRP >0.5mg/dL | Piper Fatigue Scale | 42% of patients with ID were fatigued v 23% without ID (p = 0.06).  Multiple logistic regression for ID as risk factor for fatigue: OR 2.1 (CI 0.8-5.8), p=0.13 | Tendency to fatigue but not statistically significant |
| Aluzuite *et al,* 2019(11) | Adult IBD: >18 years | 113  UC: 43 CD: 79 | Prospective, observational, cross-sectional, single centre, outpatients | Determine prevalence of fatigue in IBD patients and investigate associated variables. | Active (n= 70) Remission(n=43) Active = ≥1 of: CDAI>150, SCCAI>5, FCP>150,  CRP >5. 70% of CD and 49% of UC had active disease | Comparing mean fatigue scores in iron deficient v non-iron deficient patients. | ID  ID: 54  Non-ID: 59 | ID: ferritin < 30µg/L or <100µg/L in inflammation. | 1.MFI: each dimension scored 4 – 20. >10 = moderate fatigue and > 14 = severe fatigue. Overall >50: mod to severe. >70: severe fatigue  2.BFI >4 = moderate fatigue, >6 = severe fatigue | No data provided. No association between presence of ID and fatigue measures. | No |
| Bager *et al,* 2012 (12) | Adult IBD | 425  UC: 174 CD: 251 | Prospective, observational, cross-sectional, multicentre, outpatients | Prevalence of fatigue and the determinants of fatigue in IBD outpatients. | Any disease activity.  Active disease defined by HBI >4, SCCAI > 4. | Comparing mean fatigue scores in iron deficient v non-iron deficient patients. | ID  ID: 146  Non ID: 279 | ID: ferritin < 30µg/L or <100µg/L in inflammation | MFI-20: 5 dimensions of fatigue. Score of 4 – 20 in each dimension. Higher score is more fatigue | No data provided. No difference in fatigue scores in ID v non-ID. | No |

The studies in the table were all confirmed to be eligible to be included in the review. Data extraction was undertaken by SS between 16/01/2023 – 08/03/2023.

References:

1. Banovic I, Gilibert D, Jebrane A, Cosnes J. Personality and fatigue perception in a sample of IBD outpatients in remission: a preliminary study. J Crohns Colitis. 2012;6(5):571-7.

2. Chavarría C, Casanova MJ, Chaparro M, Barreiro-de Acosta M, Ezquiaga E, Bujanda L, et al. Prevalence and Factors Associated With Fatigue in Patients With Inflammatory Bowel Disease: A Multicentre Study. Journal of Crohn's and Colitis. 2019;13(8):996-1002.

3. Grimstad T, Norheim KB, Isaksen K, Leitao K, Hetta AK, Carlsen A, et al. Fatigue in Newly Diagnosed Inflammatory Bowel Disease. JOURNAL OF CROHNS & COLITIS. 2015;9(9):725-30.

4. Truyens M, De Ruyck E, Gonzales GB, Bos S, Laukens D, De Vos M. Prevalence of Fatigue and Unrecognized Depression in Patients with Inflammatory Bowel Disease in Remission under Immunosuppressants and Biologicals. J Clin Med. 2021;10(18).

5. Villoria A, García V, Dosal A, Moreno L, Montserrat A, Figuerola A, et al. Fatigue in out-patients with inflammatory bowel disease: Prevalence and predictive factors. PLoS One. 2017;12(7):e0181435.

6. Goldenberg BA, Graff LA, Clara I, Zarychanski R, Walker JR, Carr R, et al. Is iron deficiency in the absence of anemia associated with fatigue in inflammatory bowel disease? The American journal of gastroenterology. 2013;108(9):1392-7.

7. Gonzalez Alayon C, Pedrajas Crespo C, Marin Pedrosa S, Benitez JM, Iglesias Flores E, Salgueiro Rodriguez I, et al. Prevalence of iron deficiency without anaemia in inflammatory bowel disease and impact on health-related quality of life. Prevalencia de deficit de hierro sin anemia en la enfermedad inflamatoria intestinal y su impacto en la calidad de vida. 2018;41(1):22-9.

8. Herrera-Deguise C, Casellas F, Robles V, Navarro E, Borruel N. Iron deficiency in the absence of anemia impairs the perception of health-related quality of life of patients with inflammatory bowel disease. Inflammatory Bowel Diseases. 2016;22(6):1450-5.

9. Jonefjall B, Simren M, Lasson A, Ohman L, Strid H. Psychological distress, iron deficiency, active disease and female gender are independent risk factors for fatigue in patients with ulcerative colitis. United European gastroenterology journal. 2018;6(1):148-58.

10. Konig P, Jimenez K, Saletu-Zyhlarz G, Mittlbock M, Gasche C. Iron deficiency, depression, and fatigue in inflammatory bowel diseases. Eisenmangel, Depression und Erschopfung bei CED. 2020;58(12):1191-200.

11. Kis AM, Carnes M. Detecting Iron Deficiency in Anemic Patients with Concomitant Medical Problems.

12. Bager P, Befrits R, Wikman O, Lindgren S, Moum B, Hjortswang H, et al. Fatigue in out-patients with inflammatory bowel disease is common and multifactorial. Alimentary Pharmacology & Therapeutics. 2012;35(1):133-41.
